# Multimodal neuromonitoring-related complications in patients with severe acute brain injury: a retrospective cohort study

**DOI:** 10.1007/s00701-026-06950-z

**Published:** 2026-06-13

**Authors:** Tobias Sjørslev Bodilsen, Trine Hjorslev Andreasen, Markus Harboe Olsen, Anne-Sophie Worm Fenger, Alexandra Vassilieva, Helene Ravnholt Jensen, Kirsten Møller

**Affiliations:** 1 Copenhagen Neuroanaesthesiology and Neurointensive Care Research Group (CONICA), Department of Neuroanaesthesiology, The Neuroscience Centre, Copenhagen University Hospital – Rigshospitalet, Copenhagen, Denmark; 2 Department of Neurosurgery, The Neuroscience Centre, Copenhagen University Hospital – Rigshospitalet, Copenhagen, Denmark; 3 Department of Clinical Medicine, Faculty of Health and Medical Sciences, University of Copenhagen, Copenhagen, Denmark

**Keywords:** Multimodal neuromonitoring, Complications, Safety, Acute brain injury, PbtO_2_, Cerebral microdialysis

## Abstract

**Background:**

Invasive multimodal neuromonitoring (MMNM) is standard of care in many neuro-intensive care units for patients with severe acute brain injury. MMNM is used for monitoring of early signs of secondary brain injury. Still, the safety of this approach is only partly clarified. This study aimed to investigate the prevalence and types of MMNM-related complications.

**Methods:**

For this single-centre retrospective study, we included all patients admitted to the Neurointensive Care Unit at Rigshospitalet from March 2017 to December 2023, who were monitored using MMNM probes in the brain, i.e. monitoring of intracranial pressure (ICP) in combination with brain tissue oxygen tension (PbtO_2_) and/or cerebral microdialysis (CMD). Medical records and computed tomography (CT) or magnetic resonance imaging (MRI) of the head were reviewed for MMNM-related complications and functional outcome at 6 months after ictus.

**Results:**

A total of 223 patients with severe acute brain injury underwent monitoring with MMNM. Imaging was done after insertion in 196 patients (88%) of all who received MMNM. In these, haemorrhage related to the insertion was observed in 25 patients (13%), suboptimal location in 70 (36%), bone fragments in 25 (13%), and pneumocephalus in 71 (36%); none of these necessitated intervention. Among the entire cohort of 223 patients, infection related to MMNM occurred in one patient (0.4%); malfunction was reported by clinicians in 64 patients (29%) and verified in 26 (12%). After MMNM removal, 162 patients (73%) had a CT or MRI scan, of whom 11 patients (6.8%) had a new parenchymal haemorrhage; one patient (0.4%) underwent surgical haematoma evacuation. Six months functional outcome did not differ between patients with and those without MMNM-related complications.

**Conclusion:**

In this retrospective study, a high number of MMNM-related complications was found, of which the vast majority did not cause clinical concern and did not affect patient treatment or outcomes.

**Supplementary Information:**

The online version contains supplementary material available at 10.1007/s00701-026-06950-z.

## Introduction

Severe acute brain injury, such as traumatic brain injury (TBI), aneurysmal subarachnoid haemorrhage (SAH) and spontaneous intracerebral haemorrhage (ICH), is associated with high morbidity and mortality [7, 13, 24]. These patients usually require admission to an intensive care unit (ICU) with sedation and mechanical ventilation in the acute phase. However, sedation may hinder clinical assessment and detection of neurological worsening. Various combinations of multimodal neuromonitoring (MMNM) are used in Neuro-ICUs worldwide to detect and treat early signs of secondary brain injury, which is associated with unfavourable functional outcome [12, 17, 22]. Some modalities are invasive, requiring a burr hole in the skull to insert catheters for, e.g., monitoring of intracranial pressure (ICP), brain tissue oxygen tension (PbtO_2_) as well as cerebral microdialysis (CMD) [9]. The procedure incurs a risk of haemorrhage, infection, and suboptimally located catheters, the prevalence and consequences of which are only partially clarified [1, 3, 5, 10, 21].

The aim of this retrospective, single-centre, cohort study of patients with severe acute brain injury was to (1) investigate the prevalence and type of complications due to MMNM, (2) explore potential clinical risk factors of different types of complications, and (3) evaluate the relationship between MMNM-related complications and functional outcome. In accordance with the clinical practice in our institution, MMNM was defined as simultaneous use of at least two of the following modalities: ICP monitoring, PbtO_2_ monitoring, and CMD.

## Methods

In this single-centre, retrospective cohort study, we included all patients with severe acute brain injury who were admitted to the Neuro-ICU at Copenhagen University Hospital–Rigshospitalet, Denmark from March 2017 to December 2023 and monitored with at least two of the following modalities: ICP, PtbO_2_, and CMD. Complications related to external ventricular drain (EVD) was not included. The study was approved by the Centre of Regional Development, the Capital Region of Denmark (ref. R-23052162, 20 September 2023), who waived the need for written consent from patients for this type of study according to the Danish Health Act §46 and §47.

### Indications, MMNM placement and devices

During the study period, the local guidelines recommended MMNM with ICP, PbtO_2_ and CMD for patients admitted with TBI or SAH and a Glasgow Coma Scale (GCS) score < 9. Patients with ICH who were included in the ongoing KETA-BID trial [2] were also eligible to receive MMNM for research purposes. The local guidelines further recommended that MMNM be placed contralaterally to the most injured hemisphere as detected on the last CT scan before surgery in patients with TBI, and ipsilaterally to the ruptured aneurysm or, in case of a midline aneurysm, in the left hemisphere in patients with SAH. In ICH patients, the MMNM was intended to be placed ipsilaterally to the haemorrhage. However, the insertion of MMNM as well as the side of insertion ultimately depended on the neurosurgeon’s discretion and technical feasibility. Insertion of MMNM was done either in the operating room or bedside in the Neuro-ICU. The surgical procedure was performed under general combined with local anaesthesia, using a point incision followed by creation of a burr hole with a manual drill. Next, the dura was penetrated, and a bolt was inserted into the skull. Finally, the catheters were inserted intraparenchymally through the bolt.

In the period from March 2017 to June 2023, ICP monitoring was done using an intraparenchymal transducer, Codman Microsensor® ICP Transducer (Integra® Lifesciences, New Jersey, United States) connected to Codman® ICP Express™ monitor (Integra® Lifesciences, New Jersey, United States). PbtO_2_ monitoring was done using a Licox® Brain Tissue Oxygen Monitor Complete Probe Kit (Integra LifeSciences, New Jersey, United States) connected to Licox® PbtO_2_ Monitor (Integra LifeSciences, New Jersey, United States). These devices were linked through a double bolt (IM2SEU) or triple bolt (IM3SEU).

From June 2023 to December 2023 a Bolt kit PTO 2L (Raumedic, Helmbrechts, Germany) was used with a Neurovent-PTO 2L Bolt (Raumedic, Helmbrechts, Germany) for ICP, PbtO_2_, Brain tissue temperature.

Throughout the period, CMD monitoring was carried out using a 70 MD Bolt Catheter (length, 130 mm, diameter, 0.9 mm; M Dialysis AB, Stockholm, Sweden).

### Data collection and definitions

Demographic data and information on diagnosis, duration of MMNM, and Neuro-ICU and hospital lengths of stay were collected from medical records. Two authors (TSB and THA) independently assessed computed tomography (CT) scans and magnetic resonance imaging (MRI) of the head for complications as well as using the neuroradiologists’ reports. Any disagreement was resolved by consensus. All CT and MRI scans were performed only when clinically indicated. A priori, we defined the following as surgical complications: intracranial haemorrhage, bone fragments, and pneumocephalus regardless of size, and suboptimal catheter location after insertion. Excluding catheter location, surgical complications were further categorised as occurring after catheter insertion or after catheter removal. Surgical complications after removal were only noted if they were new compared to after catheter insertion. The following other events related to MMNM were collected from the medical records: dislodgement, infection that could be related to an MMNM device, leakage and catheter malfunction. Surgical complications after insertion and any other events are collectively referred to as MMNM-related complications. Additionally, MMNM-related complications requiring interventions were noted.

#### Intracranial haemorrhage 

Intracranial haemorrhage was subcategorised as subdural (SDH) or intracerebral (ICH). The maximal width of SDH was measured. ICH after catheter insertion was further classified as Grade I (< 1 ml), Grade II (1 ml) or Grade III (> 1 ml) for haematoma located along or at the tip of the catheter [1]. ICH volume was calculated using the ABC/2 formula, based on maximal measurements in centimetres in the A, B, and C planes obtained from multiplanar reformations [4].

#### Infection

Skin infection was defined as clinical signs of infection with or without a positive bacterial culture more than 48 h after MMNM insertion, in combination with administration of antibiotics on this indication. Intracranial infection was diagnosed from the medical records if intrathecal and/or systemic antibiotics were prescribed, and suspected or verified intrathecal infection due to MMNM was noted as the indication. Catheters were only sent for culture when clinically indicated.

#### Catheter location

Optimal catheter location was defined if the tip of the catheter was visible on CT in normally appearing subcortical parenchyma, as intended, in the frontal lobe without touching other intracerebral devices. Suboptimal catheter location was therefore defined as a catheter located elsewhere, further subcategorised as extraaxial, in the grey matter, within a lesion, perilesional, intraventricular, paraventricular, or in the midline, or if the catheter was kinked or the tip appeared to be in physical contact with e.g. an extra ventricular drain.

#### Catheter malfunction

A malfunction was considered to be verified if it was confirmed by a clinician trained to interpret data from MMNM with or without functional validation. Functional validation of PbtO_2_ catheter was done by increasing the fraction of inspired oxygen (FiO_2_) to 100% for minimum of 5 min. If PbtO_2_ did not rise substantially within 10 min during the oxygen increase, despite an increase in peripheral oxygen saturation, the probe was deemed to be non-functioning. CMD malfunction was defined by absence of fluid in the microdialysis chamber. A non-verified malfunction was defined as a report of catheter malfunction entered in the medical record that was not validated by a clinician who had received specific training in MMNM.

#### Interventions

Interventions were defined as reinsertion of catheter and/or bolt, including reinsertion through a new burr hole, and surgery or removal of MMNM equipment due to any MMNM-related complication.

#### Functional outcome

Functional outcome at 6 months was recorded using the Glasgow Outcome Scale-Extended (GOSE) and modified Rankin Scale (mRS) by two researchers independently and collected by reviewing medical records. An unfavourable functional outcome was defined as GOSE 1–5 and mRS 3–6.

All data were entered and stored in REDCap (Version LTS 13.7.14 Vanderbilt University, Tennessee, United States of America).

### Statistical analyses

Analyses were performed using the statistical software R (R version 4.5.0, R Core Team 2024, Vienna, Austria). Continuous variables were assessed for normal distribution with the Shapiro–Wilk test and visually with QQ plots. If normally distributed, data was presented as the mean and standard deviation (SD); otherwise, the median and interquartile range (IQR) were reported. Categorical variables were reported as numbers (*n*) and percentages (%). The Wilcoxon test and Fisher’s exact test were performed to compare complications between groups. Logistic regression was performed to investigate odds ratio (OR) for selected complications. A *p*-value < 0.05 was considered statistically significant.

## Results

### Patient characteristics and MMNM-related procedures

Over an almost 7-year period, 223 patients were monitored with MMNM, of whom 6 were under 18 years of age (Table 1; for comorbidities and Neuro-ICU infection, please refer to Supplementary Table 1 and for severity scoring of patients with SAH, please refer to Supplementary Table 2). Median time from admission to MMNM insertion was 0 days (IQR: 0–1). Insertion of MMNM was performed in the operating room in 188 patients (84%), and bedside in the Neuro-ICU in 35 patients (16%). Seventy-six of 117 patients (65%) with TBI underwent craniotomy or craniectomy with haematoma evacuation. In SAH patients, craniotomy was performed in 35 of 102 cases (34%), one of which was combined with endovascular treatment; 54 patients (53%) were treated endovascularly only, and treatment data were missing for 13 patients. All 4 patients with ICH underwent a craniotomy with haematoma evacuation. A total of 196 patients (88%) had a CT scan performed (median of 2 days, IQR: 1–4) after insertion. Reinsertion of one or more catheters was done for 37 patients (17%), of whom six needed a second reinsertion; a total of 43 reinsertions were performed of which 25 (58%) were followed by a CT scan. MMNM equipment was removed in part or in full and without reinsertion in 43 patients (19%) due to an MMNM-related complication. MMNM modalities and the anatomical location of MMNM devices are summarized in Table 2 and 3.

**Table 1:** Patient characteristics

	Overall	No MMNM complications	MMNM complications	*P*-value*
	(*N* = 223)	(*N* = 58)	(*N* = 165)	
Demographics				
Female	109 (49%)	34 (59%)	75 (46%)	0.09
Age (years)	55 [40–65]	57 [40–64]	53 [39–65]	0.70
Diagnosis				
ICH	4 (1.8%)	1 (1.7%)	3 (1.8%)	0.20
SAH	102 (46%)	32 (55%)	70 (42%)	
TBI	117 (53%)	25 (43%)	92 (56%)	
Comorbidities^$^	74 (33%)	21 (36%)	53 (32%)	0.63
Admission				
Initial GCS	6 [3–12]	6 [3–12]	6 [3–12]	0.69
In-hospital GCS	3 [3–10]	3 [3–12]	3 [3–9]	0.29
Charlson score	1 [0–3]	1 [0–3]	1 [0–3]	0.98
APACHE II score	21 [18–25]	21 [18–25]	22 [18–25]	0.69
External ventricular drainage	102 (46%)	21 (36%)	81 (49%)	0.09
Combi-drain	99 (44%)	30 (52%)	69 (42%)	0.22
Duration of MMNM (days)	10 [6–16]	9 [3–16]	11 [6–17]	0.05
Length of ICU stay (days)	16 [6–29]	14 [5–28]	16 [8–29]	0.24
Length of hospital stay (days)	46 [15–88]	48 [18–90]	46 [15–88]	0.95
Infection^$^	161 (72%)	39 (67%)	122 (74%)	0.39
Glasgow Outcome Scale-Extended				
Unfavourable outcome (1–5)	166 (74%)	41 (71%)	125 (76%)	0.60
Favourable outcome (6–8)	48 (21%)	14 (24%)	34 (20%)	
Missing	9 (4.0%)	3 (5.2%)	6 (3.6%)	
Modified Rankin Scale				
Unfavourable outcome (3–6)	152 (68%)	39 (67%)	113 (68%)	0.88
Favourable outcome (0–2)	62 (28%)	16 (28%)	46 (28%)	
Missing	9 (4.0%)	3 (5.2%)	6 (3.6%)	
6 months mortality				
Dead	68 (31%)	15 (26%)	53 (32%)	0.48
Alive	150 (67%)	41 (71%)	109 (66%)	
Missing	5 (2.2%)	2 (3.4%)	3 (1.8%)	

Numbers are count (percentage) or median [IQR]

*APACHE* acute physiology and chronic health evaluation; Combi-drain, external ventricular drainage draining cerebrospinal fluid and measuring intracranial pressure simultaneously, *GCS* Glasgow Coma Scale, *ICH* Intracerebral haemorrhage, *ICU* intensive care unit, *MMNM* multimodal neuromonitoring, *SAH* subarachnoid haemorrhage, *TBI* traumatic brain injury

*Fisher’s test for categorical variables, Wilcoxon’s test for continuous variables

^$^ Please see supplement 1 for more details

**Table 2:** MMNM modalities used (*N* = 223)

Modalities	*N* (%)
PbtO_2_ and ICP*	6 (2.7%)
PbtO_2_ and ICP	33 (15%)
PbtO_2_ and CMD	96 (43%)
PbtO_2_, ICP and CMD	71 (32%)
PbtO_2,_ ICP, Tbt and CMD	17 (7.6%)

*CMD* cerebral microdialysis, *ICP* intracranial pressure, *PbtO*_2_ brain tissue oxygen tension, *Tbt* Brain temperature

*In 6 patients, no ICP catheter was inserted through the double bolt (IM2SEU) due to surgical difficulties, and contrary to the intention. Thus, ICP was measured using an EVD combi-drain or a tunnelled ICP catheter, and only the PbtO2

**Table 3:** Anatomical location of MMNM equipment by clinical diagnosis (*N* = 223)*

Condition	Hemisphere location	*N* (%) of patients
SAH (non-midline aneurysm)	Contralateral hemisphere	44 (20%)
	Ipsilateral hemisphere	44 (20%)
SAH (midline aneurysm)	Right hemisphere	8 (3.5%)
	Left hemisphere	3 (1.3%)
Aneurysm not found	Right hemisphere	2 (0.9%)
	Left hemisphere	1 (0.4%)
TBI	Most injured hemisphere	41 (18%)
	Contralateral hemisphere	76 (34%)
ICH	Contralateral hemisphere	2 (0.9%)
	Ipsilateral hemisphere	2 (0.9%)

*At first placement. Subsequent insertion generally on same side, except for three patients with TBI

*ICH* intracerebral haemorrhage, *SAH* subarachnoid haemorrhage, *TBI* traumatic brain injury

### MMNM-related complications

Table 4 shows an overview of surgical complications after primary insertion of MMNM. Haemorrhage was observed in 25 patients (13%), comprising 18 patients (9%) with subdural haematoma (median thickness, 0.4 cm (IQR 0.3–0.5)) and 9 (5%) with intracerebral haemorrhage (Grade I = 5 (2.2%), Grade II = 0, and Grade III = 4 (1.7%), maximum size 3.3 ml); none required surgical treatment. In 2 patients, haemorrhage was present both subdurally and in the parenchyma. No haemorrhage was observed after catheter reinsertion.

**Table 4:** MMNM surgery related complications after primary insertion

Complications	All probes	Integra LifeSciences			
		Double bolt (PbtO2 only*)	Double bolt	Triple bolt	Raumedic^$^
	(*N* = 196)	(*N* = 6)	(*N* = 114)	(*N* = 62)	(*N* = 14)
Haemorrhage	25 (13%)	3 (50%)	12 (101%)	9 (15%)	1 (7.1%)
ICH	9 (5%)	1 (17%)	5 (4.4%)	2 (3.2%)	1 (7.1%)
SDH	18 (9%)	2 (33%)	8 (7.0%)	8 (12.9%)	0 (0%)
Volume of ICH (ml)	0.6 [0.2–2.2]	2.7 [2.7–2.7]	0.2 [0.2–0.6]	1.1 [0.6–1.6]	1.6 [1.6–1.6]
Width of SDH (cm)	0.4 [0.3–0.5]	0.9 [0.9–0.9]	0.3 [0.3–0.4]	0.4 [0.3–0.5]	-
Suboptimal catheter placement	70 (36%)	4 (67%)	38 (33%)	24 (39%)	4 (29%)
Bone fragments	25 (13%)	2 (33%)	12 (11%)	6 (10%)	5 (36%)
Pneumocephalus	71 (36%)	2 (33%)	40 (35%)	26 (42%)	3 (21%)

Numbers are count (percentage) or median (IQR)

*ICH* intracerebral haemorrhage, *IQR* inter-quartile range, *MMNM* multimodal neuromonitoring, *SDH* subdural haemorrhage

*In 6 patients, no ICP catheter was inserted through the double bolt (IM2SEU) due to surgical difficulties, and contrary to the intention. Thus, ICP was measured using an EVD combi-drain or a tunnelled ICP catheter, and only the PbtO2

^$^ Neurovent-PTO 2L Bolt

One or more catheters were found to be suboptimally located after first insertion in 70 patients (36%) (sub-categories are found in Supplementary Table 3). Eight out of 37 (22%) and 2 out of 6 (33%) patients, respectively, had suboptimally located catheters after the first and second reinsertion.

Malfunction of one or more catheters was one of the most common complications with 26 of 62 (42%) verified cases; however, a suboptimal located catheter was not related to malfunction (OR for malfunction, suboptimal vs. optimal location, 0.64 (95% CI: 0.36–1.16), *p* = 0.14). Dislodgement occurred in 19 cases.

Seventy-one patients (36%) had MMNM-related pneumocephalus consisting mostly of small air bubbles along the catheters (66%) or extra-axially (34%) (OR for malfunction given pneumocephalus, 0.95 (95% CI: 0.53–1.72), *p* = 0.87). Only one patient had pneumocephalus on CT after a reinsertion. 

Bone fragments were detected in 25 patients (13%) after primary insertion (OR for malfunction given bone fragments, 3.7 (95% CI: 0.76–14.5), *p* = 0.19). One patient had bone fragments after a reinsertion.

Forty-five patients (20%) were treated with antibiotics for suspected intracranial infection. In one patient (0.4%), Staphylococcus haemolyticus was cultured from a PbtO_2_ monitoring catheter; the remaining infections were thought to be ventriculitis associated with the presence of an EVD, and the indication for antibiotic treatment was not given as an MMNM-related infection in the medical chart. No skin infection occurred in relation to MMNM.

**Fig. 1:**
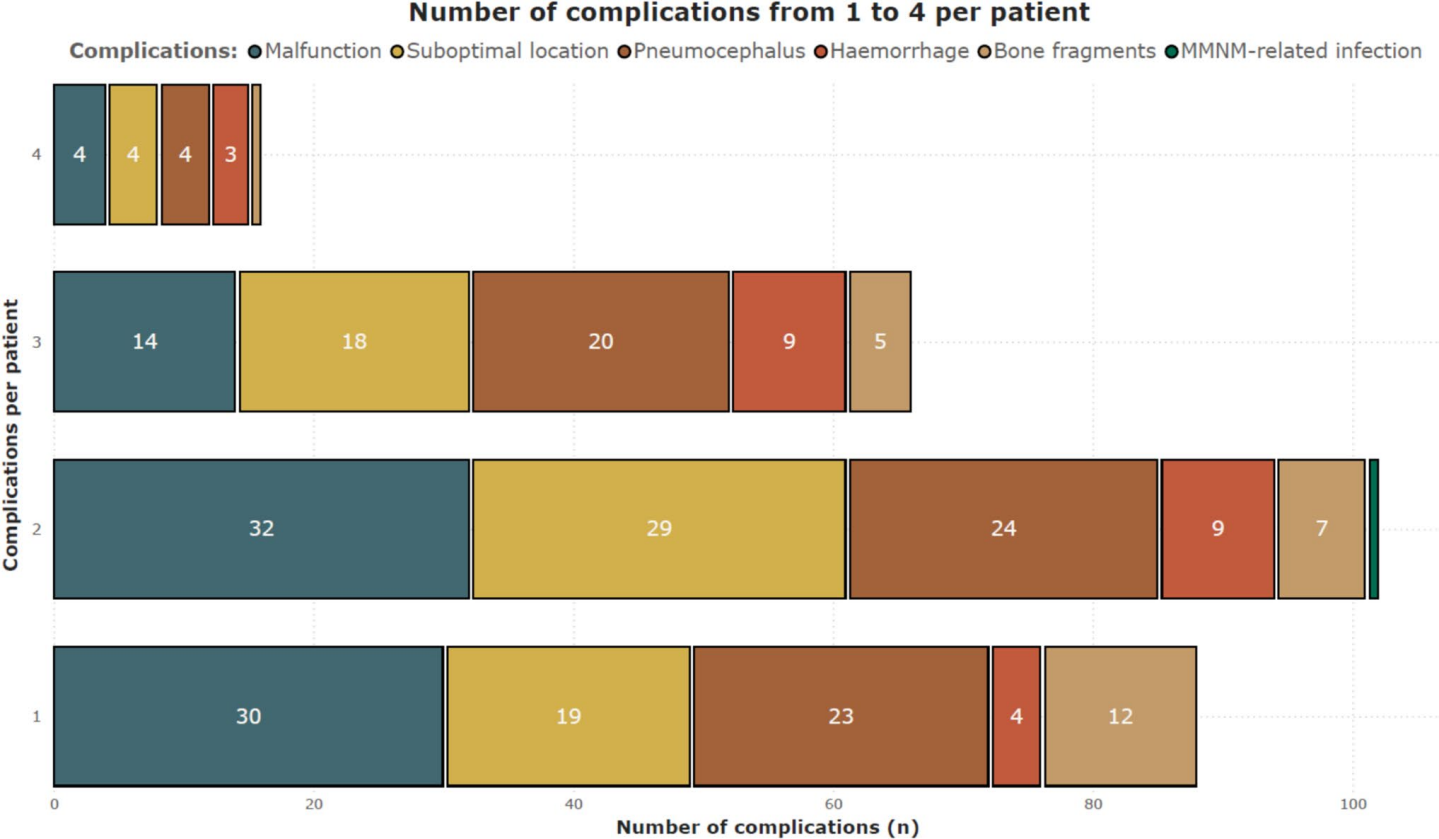
Distribution of the number of complications per patient

In total, at least one MMNM-related complication was found in 165 patients (74%) after insertion, at a total of 272 complications (Fig. 1). Subgroup comparison can be found in Supplementary Table 4 to explore potential clinical risk factors of different types of complications. These analyses showed no statistically significant differences in MMNM‑related complications between bedside and operating room insertion of MMNM.

### Complications after MMNM removal

After removal of MMNM, 162 patients (73%) underwent CT or MRI, of whom 79 patients (49%) had at least one new MMNM-related complication compared to after insertion. Thus, 11 patients (6.8%) developed a parenchymal haemorrhage related to MMNM (median volume, of 0.09 (IQR: 0.003–0.18) ml). One patient had an intracerebral haemorrhage (28 ml) which was surgically evacuated. Three patients with a parenchymal haemorrhage did not have a CT scan after MMNM insertion. One patient developed an SDH after removal. Lastly, 66 patients (40%) had bone fragments, of whom 12 did not have a CT scan after insertion, and 13 patients (8%) had some degree of pneumocephalus, of whom 3 did not have a CT scan after insertion.

### Association between MMNM complications and patient outcomes

No difference in 6 months functional outcome or mortality was found between patients with and those without complications related to MMNM (Table 1). Unfavourable functional outcome on the mRS scale appeared in 68% of patients with MMNM-related complications compared to 67% of those without, and 76% of patients with MMNM-related complications compared to 71% of those without had unfavourable GOSE outcome. Six months mortality was 32% and 26%, for patients with and those without MMNM-related complications, respectively.

## Discussion

MMNM is standard of care in many Neuro-ICUs for patients with severe acute brain injury and is generally considered safe with complication rates consistent with other intracranial devices used for these types of patients. Using our definition of MMNM-related complications, we found a high overall complication rate, with 165 patients (74%) having at least one complication, most of which were attributed to malfunction, suboptimally located catheters, and pneumocephalus. However, the vast majority were trivial, did not require interventions, and did not affect patient treatment; only one patient required surgical treatment due to a haematoma occurring after removal of MMNM. Importantly, MMNM-related complications were not associated with unfavourable functional outcome or mortality after 6 months.

### Haemorrhage

We observed 25 patients (13%) with haemorrhage after MMNM insertion, which is within the previously reported range from 0–40% [5, 10]. Notably, this is also within the range of EVD-related haemorrhage ranging from 0–41% [16, 19]. Few publications report the need for surgical treatment of MMNM-related haemorrhage [1, 3, 5, 15]. In our cohort, one patient required surgery for removal of an intracerebral haematoma that developed after the MMNM equipment was removed. This corresponds to previous reports, where most haemorrhages were clinically nonsignificant compared to the primary brain injury and did not require intervention [5, 15].

### Suboptimally located catheters and malfunction

In our cohort, one of every three catheters were suboptimally located, which is high compared with other studies, with a reported rate ranging from 3–14% [1, 3, 10, 21]. We pre-specified a rather wide definition of suboptimally located catheters with nine sub-categories, many of which have not previously been reported elsewhere in the literature. This may have contributed to the lack of an association with malfunction; thus, despite our findings we continue to believe that careful positioning of MMNM devices by trained neurosurgeons is important to obtain clinical benefit.

Malfunction was reported for one out of four catheters, but close to 60% of these were non-verified, meaning that it was not validated by an MMNM-trained clinician. For comparison, the literature reports rates of malfunction between 5–43% [1, 3, 21]. As a limitation of our retrospective study design, the specific causes of catheter malfunctioning were difficult to categorise. Besides functional testing of the equipment, distinguishing between correctly functioning and malfunctioning equipment mainly depends on the training and experience of the clinician. Bailey et al. [3] reported that 5% of all PbtO_2_ catheters were replaced on suspicion of malfunctioning, but that 54% of these were confirmed to be functioning normally, which corroborates the need for adequate training of the staff.

### Bone fragments and pneumocephalus

Few reports have reported the prevalence of pneumocephalus and bone fragments in brain parenchyma after insertion or removal of MMNM, let alone their clinical consequences [1, 5, 10, 11]. When reported, they are usually categorised as clinically asymptomatic. In our study, none of these phenomena were documented in the patient records or treated, and no clinical consequences were observed. The long-term clinical significance of bone fragments and pneumocephalus, however, remains unclear. Bone fragments were more prevalent after MMNM removal, probably due to obscuring artefacts while bolts and catheters were still in place.

### Infection

In our study cohort, one patient was treated for an MMNM-related infection; others have reported prevalences between 0–5% [1, 3, 10, 21]. Although it can be challenging to determine whether central nervous system (CNS) infections originate from MMNM or EVD, the risk of MMNM-related infections seems low compared to, e.g. that of EVD-related CNS infections, the prevalence of which ranges from 0–28% [16].

Patients with severe acute brain injury, which inherently has a high morbidity and mortality, may benefit from MMNM as it provides crucial information on the ongoing pathophysiology and potential risk of secondary brain injury [7, 12, 14, 17, 18]. MMNM gives clinicians the opportunity to personalise treatment according to the patient’s current condition. Although results from the BOOST-3 [6] and the BONANZA [23] trials in patients with TBI are still awaited, published studies suggest that combined PbtO_2_- and ICP- guided therapy may improve outcome [12, 17, 18] in those with high ICP [20].

### Strengths and limitations

The major strength of this study was the large cohort of patients with severe acute brain injury, both TBI and non-TBI. Additionally, two authors independently assessed CT and MRI scans, and two separate authors independently assessed functional outcome. However, this study also has limitations. Because it was a single-centre retrospective study, the external validity remains limited; though, the findings mostly reflected previous studies. A surgical complication scale, such as the Clavien Dindo Scale of Surgical Complications [8] could have been useful for comparison and external validation. Nevertheless, this scale is intended for more general elective surgery, and to our knowledge no similar scale has been devised for emergency surgery. Next, the lack of formal radiology training of the authors in reviewing head CTs or MRIs is a limitation as this could lead to over- or underestimation of the frequency of surgical complications. It would have been more appropriate, but also resource-demanding to have a radiologist review the head CTs and MRIs. Another limitation of the study is that a postoperative CT scan was not performed systematically as standard of care, but only on clinical indication, which could lead to underestimation of the surgical complications found on the CT scans after insertion. Lastly, we did not assess the level of experience of neurosurgeons performing the procedure in the analysis, nor did we compare complications occurring during daytime or night shifts.

## Conclusion

We found a high rate of MMNM-related complications. Most were believed to be MMNM-related findings rather than true complications, as they did not cause clinical concern, with very few requiring interventions. We found no association between MMNM-related complications and functional outcome or mortality after 6 months. MMNM appears generally safe to patients with severe acute brain injury. However, greater knowledge of MMNM-guided therapy on patient outcome is required to support the current use of MMNM.

## Supplementary Information

Below is the link to the electronic supplementary material.

ESM 1 Additional supporting information is available online in the Supplementary Materials. Supplementary Table 1: Subgroups of comorbidities and Neuro-ICU infection. Supplementary 2: Severity scoring of patients with SAH. Supplementary Table 3: Nine subgroups of suboptimal located catheters following the initial insertion of MMNM. Supplementary 4: MMNM-related complications categorized for comparative analysis to identify potential clinical risk factors associated with different complication types.  (DOCX 39.5 KB)

## Data Availability

No datasets were generated or analysed during the current study.

## Abbreviations

CSF: Cerebrospinal fluid
CT: Computed tomography
EVD: External ventricular drain
GCS: Glasgow Coma Scale
ICH: Intracerebral haemorrhage
ICP: Intracranial pressure
ICU: Intensive care unit
MMNM: Multimodal neuromonitoring
MRI: Magnetic resonance imaging
SAH: Subarachnoid haemorrhage
TBI: Traumatic brain injury

## References

[1] Al Barajraji M, Bogossian E, Dewitte O, Gaspard N, El Hadwe S, Minini A, Andre J, Taccone FS, Schuind S, Barrit S (2021) Safety profile of an intracranial multimodal monitoring bolt system for neurocritical care: a single-center experience. Acta Neurochir (Wien) 163(12):3259–3266 34495407 10.1007/s00701-021-04992-z

[2] Andreasen TH, Olsen MH, Gluud C, Lindschou J, Fabricius M, Hauerberg J, Møller K (2025) S-ketamine versus placebo for cortical spreading depolarisation in severe acute brain injury (KETA-BID): protocol for a pilot, randomised, blinded clinical trial. BMJ Open 15(7):e101426 40721263 10.1136/bmjopen-2025-101426 PMC12306330

[3] Bailey RL, Quattrone F, Curtin C, Frangos S, Maloney-Wilensky E, Levine JM, LeRoux PD (2019) The safety of multimodality monitoring using a triple-lumen bolt in severe acute brain injury. World Neurosurg. Elsevier Inc., pp e62–e67 10.1016/j.wneu.2019.05.195 31195129

[4] Barras CD, Asadi H, Phal PM, Tress BM, Davis SM, Desmond PM (2016) Audit of CT reporting standards in cases of intracerebral haemorrhage at a comprehensive stroke centre in Australia. J Med Imaging Radiat Oncol 60(6):720–727 27378602 10.1111/1754-9485.12491

[5] Barrit S, El Hadwe S, Al Barajraji M, Torcida N, Bogossian EG, André J, Niset A, Carron R, Taccone FS, Madsen J (2024) Complications of intracranial multimodal monitoring for neurocritical care: a systematic review and meta-analysis. Neurocrit Care 40(3):1182–1192 37991675 10.1007/s12028-023-01885-0

[6] Bernard F, Barsan W, Diaz-Arrastia R, Merck LH, Yeatts S, Shutter LA (2022) Brain oxygen optimization in severe traumatic brain injury (BOOST-3): a multicentre, randomised, blinded-endpoint, comparative effectiveness study of brain tissue oxygen and intracranial pressure monitoring versus intracranial pressure alone. BMJ Open 12(3):e060188 35273066 10.1136/bmjopen-2021-060188 PMC8915289

[7] Claassen J, Park S (2022) Spontaneous subarachnoid haemorrhage. Lancet 400(10355):846–862 35985353 10.1016/S0140-6736(22)00938-2 PMC9987649

[8] Dindo D, Demartines N, Clavien PA (2004) Classification of surgical complications: a new proposal with evaluation in a cohort of 6336 patients and results of a survey. Ann Surg 240(2):205–213 15273542 10.1097/01.sla.0000133083.54934.ae PMC1360123

[9] Falcone JA, Chen JW (2023) Technical notes on the placement of cerebral microdialysis: a single center experience. Front Neurol. 10.3389/fneur.2022.1041952 36698903 10.3389/fneur.2022.1041952 PMC9868911

[10] Foreman B, Ngwenya LB, Stoddard E, Hinzman JM, Andaluz N, Hartings JA (2018) Safety and reliability of bedside, single burr hole technique for intracranial multimodality monitoring in severe traumatic brain injury. Neurocrit Care 29(3):469–480 29949001 10.1007/s12028-018-0551-7

[11] González I, Santamarta D (2018) Multimodal neuromonitoring catheter insertion Secondary complications. ICU Manag Pract 18(4):266–267

[12] Gouvêa Bogossian E, Diosdado A, Barrit S, Al Barajraji M, Annoni F, Schuind S, Taccone FS (2022) The impact of invasive brain oxygen pressure guided therapy on the outcome of patients with traumatic brain injury: a systematic review and meta-analysis. Neurocrit Care 37(3):779–789 36180764 10.1007/s12028-022-01613-0

[13] Gross BA, Jankowitz BT, Friedlander RM (2019) Cerebral Intraparenchymal Hemorrhage: A Review. JAMA - Journal of the American Medical Association 321(13):1295–1303 10.1001/jama.2019.2413 30938800

[14] Hoh BL, Ko NU, Amin-Hanjani S et al (2023) 2023 guideline for the management of patients with aneurysmal subarachnoid hemorrhage: a guideline from the American Heart Association/American Stroke Association. Stroke 54(7):E314–E370 37212182 10.1161/STR.0000000000000436

[15] Kieninger M, Meichelböck K, Bele S, Bründl E, Graf B, Schmidt NO, Schebesch KM (2021) Brain multimodality monitoring in patients suffering from acute aneurysmal subarachnoid hemorrhage: clinical value and complications. Neurosignals 20(3):703–710 10.31083/j.jin2003075 34645104

[16] Lele AV, Hoefnagel AL, Schloemerkemper N, Wyler DA, Chaikittisilpa N, Vavilala MS, Naik BI, Williams JH, Raghavan LV, Koerner IP (2017) Perioperative management of adult patients with external ventricular and lumbar drains: guidelines from the society for neuroscience in anesthesiology and critical care. J Neurosurg Anesthesiol 29(3):191–210 28169966 10.1097/ANA.0000000000000407

[17] Le Roux P, Menon DK, Citerio G et al (2014) Consensus summary statement of the International Multidisciplinary Consensus Conference on Multimodality Monitoring in Neurocritical Care: a statement for healthcare professionals from the Neurocritical Care Society and the European Society of Intensive Care Medicine. Neurocrit Care 21(2):1–26 10.1007/s12028-014-0041-5 PMC10596301 25208678

[18] Okonkwo DO, Shutter LA, Moore C et al (2017) Brain oxygen optimization in severe traumatic brain injury phase-II: a phase II randomized trial. Crit Care Med 45(11):1907–1914 29028696 10.1097/CCM.0000000000002619 PMC5679063

[19] Ortolano F, Carbonara M, Stanco A, Civelli V, Carrabba G, Zoerle T, Stocchetti N (2017) External ventricular drain causes brain tissue damage: an imaging study. Acta Neurochir (Wien) 159(10):1981–1989 28791520 10.1007/s00701-017-3291-0

[20] Payen J-F, Launey Y, Chabanne R et al (2023) Intracranial pressure monitoring with and without brain tissue oxygen pressure monitoring for severe traumatic brain injury in France (OXY-TC): an open-label, randomised controlled superiority trial. Lancet Neurol 22(11):1005–1014 37863590 10.1016/S1474-4422(23)00290-9

[21] Stuart RM, Schmidt M, Kurtz P et al (2010) Intracranial multimodal monitoring for acute brain injury: a single institution review of current practices. Neurocrit Care 12(2):188–198 20107926 10.1007/s12028-010-9330-9

[22] Tas J, Czosnyka M, van der Horst ICC, Park S, van Heugten C, Sekhon M, Robba C, Menon DK, Zeiler FA, Aries MJH (2022) Cerebral multimodality monitoring in adult neurocritical care patients with acute brain injury: a narrative review. Front Physiol. 10.3389/fphys.2022.1071161 36531179 10.3389/fphys.2022.1071161 PMC9751622

[23] Udy AA, Jeffcote T, Battistuzzo CR et al (2025) Brain tissue oxygen monitoring for severe traumatic brain injury: the international multicentre randomised controlled BONANZA-GT study protocol. BMJ Open 15(10):e106962 41043845 10.1136/bmjopen-2025-106962 PMC12496108

[24] Zhou Y-T, Tong D-M, Wang S-D, Ye S, Xu B-W, Yang C-X (2018) Acute spontaneous intracerebral hemorrhage and traumatic brain injury are the most common causes of critical illness in the ICU and have high early mortality. BMC Neurol 18(1):127 10.1186/s12883-018-1127-z 30149796 10.1186/s12883-018-1127-z PMC6112133

