# Supplementary material for: Multimodal neuromonitoring-related complications in patients with severe acute brain injury: a retrospective cohort study
Source: Acta Neurochir (Wien). 2026 Jun 13;168(1):190. doi: 10.1007/s00701-026-06950-z (PMC13427960; doi:10.1007/s00701-026-06950-z)
Supplement: Supplementary file 1 — Additional supporting information is available online in the Supplementary Materials. Supplementary Table 1: Subgroups of comorbidities and Neuro-ICU infection. Supplementary 2: Severity scoring of patients with SAH. Supplementary Table 3: Nine subgroups of suboptimal located catheters following the initial insertion of MMNM. Supplementary 4: MMNM-related complications categorized for comparative analysis to identify potential clinical risk factors associated with different complication types. (DOCX 39.5 KB) [file 701_2026_6950_MOESM1_ESM.docx]

**Multimodal neuromonitoring-related complications in patients with severe acute brain injury: a retrospective cohort study**

**Authors**

Tobias Sjørslev Bodilsen^1^, Trine Hjorslev Andreasen^1,2^, Markus Harboe Olsen^1^, Anne-Sophie Worm Fenger^1^, Alexandra Vassilieva^1^, Helene Ravnholt Jensen^1^*,* Kirsten Møller^1,3^

**Affiliation**

^1^ Copenhagen Neuroanaesthesiology and Neurointensive Care Research Group (CONICA). Department of Neuroanaesthesiology, The Neuroscience Centre, Copenhagen University Hospital – Rigshospitalet, Copenhagen, Denmark

^2^ Department of Neurosurgery, The Neuroscience Centre, Copenhagen University Hospital – Rigshospitalet, Copenhagen, Denmark

^3^ Department of Clinical Medicine, Faculty of Health and Medical Sciences, University of Copenhagen, Denmark

**Corresponding author**

Tobias Sjørslev Bodilsen, MD

Department of Neuroanaesthesiology, The Neuroscience Centre, Copenhagen University Hospital – Rigshospitalet, Copenhagen, Denmark

Blegdamsvej 9, 2100 Copenhagen, Denmark

E-mail: [Tobias.sjoerslev.bodilsen.01@regionh.dk](mailto:Tobias.sjoerslev.bodilsen.01@regionh.dk)

**ORCID of authors**

Tobias Sjørslev Bodilsen: <https://orcid.org/0009-0007-7050-1985>
Trine Hjorslev Andreasen: <https://orcid.org/0000-0001-5707-8712>
Markus Harboe Olsen: <https://orcid.org/0000-0003-0981-0723>
Anne-Sophie Worm Fenger: <https://orcid.org/0000-0002-6423-4549>
Alexandra Vassilieva: <https://orcid.org/0000-0002-9476-5850>
Kirsten Møller: <https://orcid.org/0000-0003-3058-1072>

**Supplementary material**

| **Supplementary Table 1** Comorbidities and Neuro-ICU infection | | | | |
| --- | --- | --- | --- | --- |
|  | **Overall** | **No MMNM complications** | **MMNM complications** | ***P*-value*** |
|  | **(N=223)** | **(N=58)** | **(N=165)** |  |
| **Comorbidities** |  |  |  |  |
| Cardiovascular disease | 9 (4.0%) | 1 (1.7%) | 8 (4.8%) | 0.45 |
| Hypertension | 31 (14%) | 8 (14%) | 23 (14%) | 1 |
| Previously neurological disease | 12 (5.4%) | 4 (6.9%) | 8 (4.8%) | 0.51 |
| Diabetes | 13 (5.8%) | 2 (3.4%) | 11 (6.7%) | 0.52 |
| Liver disease | 4 (1.8%) | 1 (1.7%) | 3 (1.8%) | 1 |
| Chronic obstructive pulmonary disease | 11 (4.9%) | 3 (5.2%) | 8 (4.8%) | 1 |
| Anti-platelet / -coagulant treatment | 25 (11%) | 5 (8.6%) | 20 (12%) | 0.63 |
| **Infection** |  |  |  |  |
| Urinary tract infection | 33 (15%) | 10 (17%) | 23 (14%) | 0.52 |
| Pneumonia | 117 (53%) | 26 (45% | 91 (55%) | 0.22 |
| Bacteraemia / sepsis | 20 (9.0%) | 6 (10%) | 14 (8.5%) | 0.78 |
| External ventricular drainage related | 44 (20%) | 13 (22%) | 31 (19%) | 0.56 |
| MMNM related | 1 (0.4%) | 0 | 1 (0.6%) | 1 |
| Numbers are count (percentage) | | | | |
| MMNM, multimodal neuromonitoring; | | | | |
| *Fisher’s test for categorical variables | | | | |

| **Supplementary Table 2 S**everity scoring of patients with SAH | | | | |
| --- | --- | --- | --- | --- |
|  | **Overall** | **No MMNM complications** | **MMNM complications** | **P-value*** |
|  | **(N=102)** | **(N=32)** | **(N=70)** |  |
| **The World Federation of Neurosurgical Societies** | | |  |  |
| Grade 1 | 12 (11.8%) | 3 (9.4%) | 9 (12.9%) | 0.777 |
| Grade 2 | 15 (14.7%) | 6 (18.8%) | 9 (12.9%) |  |
| Grade 3 | 2 (2.0%) | 1 (3.1%) | 1 (1.4%) |  |
| Grade 4 | 17 (16.7%) | 4 (12.5%) | 13 (18.6%) |  |
| Grade 5 | 56 (54.9%) | 18 (56.3%) | 38 (54.3%) |  |
| **Hunt and Hess scale** | |  |  |  |
| Grade 1 | 6 (5.9%) | 2 (6.3%) | 4 (5.7%) | 0.883 |
| Grade 2 | 16 (15.7%) | 6 (18.8%) | 10 (14.3%) |  |
| Grade 3 | 11 (10.8%) | 3 (9.4%) | 8 (11.4%) |  |
| Grade 4 | 18 (17.6%) | 7 (21.9%) | 11 (15.7%) |  |
| Grade 5 | 36 (35.3%) | 11 (34.4%) | 25 (35.7%) |  |
| Missing | 15 (14.7%) | 3 (9.4%) | 12 (17.1%) |  |
| **Fisher scale** |  |  |  |  |
| Grade 2 | 4 (3.9%) | 2 (2.8%) | 2 (6.3%) | 0.307 |
| Grade 3 | 18 (17.5%) | 11 (15.5%) | 7 (21.9% |  |
| Grade 4 | 68 (66.0%) | 50 (70.4%) | 18 (56.3%) |  |
| Missing | 13 (12.6%) | 8 (11.3%) | 5 (15.6% |  |
| Numbers are count (percentage)  MMNM, multimodal neuromonitoring; SAH, Subarachnoid Haemorrhage | | | | |
| *Fisher’s test | | | | |

| **Supplementary Table 3** Suboptimal catheter location after first placement | | | | | |
| --- | --- | --- | --- | --- | --- |
|  |  | **Integra LifeSciences** | | |  |
|  | **All probes** | **Double bolt (PbtO2 only*)** | **Double bolt** | **Triple bolt** | **Raumedic^$^** |
|  | **(N=196)** | **(N=6)** | **(N=114)** | **(N=62)** | **(N=14)** |
| **Suboptimal catheter location** | 70 (36%) | 4 (67%) | 38 (33%) | 24 (39%) | 4 (29%) |
| Intraventricular | 10 (5.1%) | 0 (0%) | 4 (3.5%) | 6 (9.7%) | 0 (0%) |
| Paraventricular | 17 (8.7%) | 0 (0%) | 12 (11%) | 4 (6.5%) | 1 (7.1%) |
| Close to a lesion | 4 (2.0%) | 0 (0%) | 3 (2.6%) | 1 (1.6%) | 0 (0%) |
| Inside a lesion | 2 (1.0%) | 1 (17%) | 0 (0%) | 0 (0%) | 1 (7.1%) |
| Grey matter | 5 (2.6%) | 0 (0%) | 4 (3.5%) | 1 (1.6%) | 0 (0%) |
| Extra-axial | 3 (1.5%) | 1 (17%) | 2 (1.8%) | 0 (0%) | 0 (0%) |
| Tip touches the EVD | 9 (4.6%) | 0 (0%) | 5 (4.4%) | 2 (3.2%) | 2 (14%) |
| Kinked catheter | 24 (12%) | 3 (50%) | 11 (9.6%) | 10 (16%) | 0 (0%) |
| Midline | 7 (3.6%) | 0 (0%) | 4 (3.5%) | 3 (4.8%) | 0 (0%) |
| Numbers are count (percentage) | | | | | |
| EVD, external ventricular drain | | | | | |
| * In 6 patients, no ICP catheter was inserted through the double bolt (IM2SEU) due to surgical difficulties, and contrary to the intention. Thus, ICP was measured using an EVD combi-drain or a tunnelled ICP catheter, and only the PbtO2.  ^$^ Neurovent-PTO 2L Bolt | | | | | |

| **Supplementary Table 4** MMNM-related complications divided by subgroups | | | | | | | | | | | | |
| --- | --- | --- | --- | --- | --- | --- | --- | --- | --- | --- | --- | --- |
|  | **Non-TBI^$^** | **TBI** | **P-value*** | **Bedside** | **Operating room** | **P-value*** | **AP or AC** | **Non AP or -AC** | **P-value*** | **Integra LifeSciences** | **Raumedic** | **P-value*** |
|  | **(N=106)** | **(N=117)** |  | **(N=35)** | **(N=188)** |  | **(N=25)** | **(N=198)** |  | **(N=206)** | **(N=17)** |  |
| **Haemorrhage** | 9 (9%) | 16 (11%) | 0.289 | 2 (6%) | 23 (12%) | 0.384 | 2 (8%) | 23 (12%) | 0.748 | 24 (12%) | 1 (6%) | 0.701 |
| **Suboptimal catheter placement** | 35 (33%) | 35 (30%) | 0.666 | 8 (22%) | 62 (33%) | 0.321 | 11 (44%) | 59 (30%) | 0.172 | 66 (32%) | 4 (24%) | 0.592 |
| **Bone fragments** | 10 (9%) | 15 (13%) | 0.525 | 5 (14%) | 20 (11%) | 0.560 | 7 (28%) | 18 (9%) | 0.012 | 20 (10%) | 5 (29%) | 0.029 |
| **Pneumocephalus** | 21 (20%) | 50 (43%) | <0.001 | 8 (23%) | 63 (34%) | 0.242 | 7 (28%) | 64 (32%) | 0.821 | 68 (33%) | 3 (18%) | 0.280 |
| **Malfunction** | 38 (36%) | 42 (36%) | 1 | 12 (34%) | 68 (36%) | 1 | 7 (28%) | 73 (37%) | 0.508 | 75 (36%) | 5 (29%) | 0.793 |
| **Replaced due to a complication** | 12 (11%) | 25 (21%) | 0.049 | 6 (17%) | 31 (17%) | 1 | 5 (20%) | 32 (16%) | 0.577 | 36 (18%) | 1 (6%) | 0.318 |
| Numbers are count (percentage) | | | | | | | | | | | | |
| AC, anti-coagulant; AP, anti-platelet; ICH, intracerebral haemorrhage; Integra LifeSciences, double (IM2SEU) or triple (IM3SEU) bolts; MMNM, multimodal neuromonitoring; Raumedic, Neurovent-PTO 2L bolt; SDH, subdural haemorrhage; TBI, traumatic brain injury  * Fisher’s test for categorical variables ^$^ Non-TBI, patients with subarachnoid or intracerebral haemorrhage | | | | | | | | | | | | |
